# An Atypical Presentation of Sympathetic Ophthalmia in an Intact Globe Following Mechanical Fall: A Case Report and Literature Review

**DOI:** 10.3390/vision5010011

**Published:** 2021-02-21

**Authors:** Chung Shen Chean, Christina S. Lim, Periyasamy Kumar, Bharat Kapoor

**Affiliations:** 1 Department of Ophthalmology, University Hospitals of Leicester NHS Trust, Infirmary Square, Leicester LE1 5WW, UK; christina.sk.lim13@gmail.com (C.S.L.); periyasamy.kumar@uhl-tr.nhs.uk (P.K.); drbharatkapoor@yahoo.com (B.K.); 2 Department of Ophthalmology, Northampton General Hospital, Cliftonville, Northampton NN1 5BD, UK

**Keywords:** sympathetic ophthalmia, globe injury, uveitis, trauma

## Abstract

Purpose: To describe an atypical case of sympathetic ophthalmia presenting after blunt trauma causing disinsertion of the iris in an intact globe. Methods: Case report. Results: A 71-year-old lady presented to the Emergency Department following a mechanical fall. On examination, she was noted to have periocular haematoma, subconjunctival haemorrhage, hyphaema, and vitreous haemorrhage in the left eye, but there was no evidence of globe rupture. The presenting visual acuity was 6/18. As the hyphaema and vitreous haemorrhage settled, a complete loss of the iris was noted with normal fundus. She was re-admitted a month later under the medical team with urinary tract infection and reduced vision in both eyes. On examination, there was mild conjunctival injection, keratic precipitates, anterior chamber flare, 180-degree posterior synechiae, and vitritis with no fundal view of the right eye. She was diagnosed with sympathetic ophthalmia and was treated with topical and systemic corticosteroid. Her vision improved gradually with treatment and was stable at 6/6 on the right (sympathising) eye and 6/9 on the left (excited) eye at final follow-up. Conclusion: Sympathetic ophthalmia may result from non-penetrating ocular trauma. Comprehensive history of mechanism of injury and ophthalmic examination is essential so that prompt treatment can be given to improve the visual prognosis of affected patients.

## 1. Introduction

Sympathetic ophthalmia is a rare condition with an annual incidence of 0.03 per 100,000. The common causes are penetrating eye injuries and retinal surgeries [1]. There has been reports that sympathetic ophthalmia can also be caused by ocular chemical burns [2], plaque brachytherapy [3], microbial keratitis [4,5], and other surgical procedures including intraocular lens implantation [6], corneal laceration and perforation repair [7,8], resection of iridociliary tumours [9], and cyclodestructive procedures [10]. Sympathetic ophthalmia typically presents as non-necrotising bilateral granulomatous panuveitis affecting the injured (exciting) eye and the fellow (sympathising) eye weeks to years after the inciting penetrating injury or intraocular surgery. It can be associated with alopecia, poliosis, and vitiligo [2]. The exact aetiology remains unknown, but it is postulated that there is a delayed hypersensitivity to sequestered uveal antigen, which damages the outer retinal pigment epithelium layer [7]. 

In this case, we report an atypical presentation of sympathetic ophthalmia resulting from iris disinsertion in an intact globe following a mechanical fall.

## 2. Case Report

**Figure 1:**
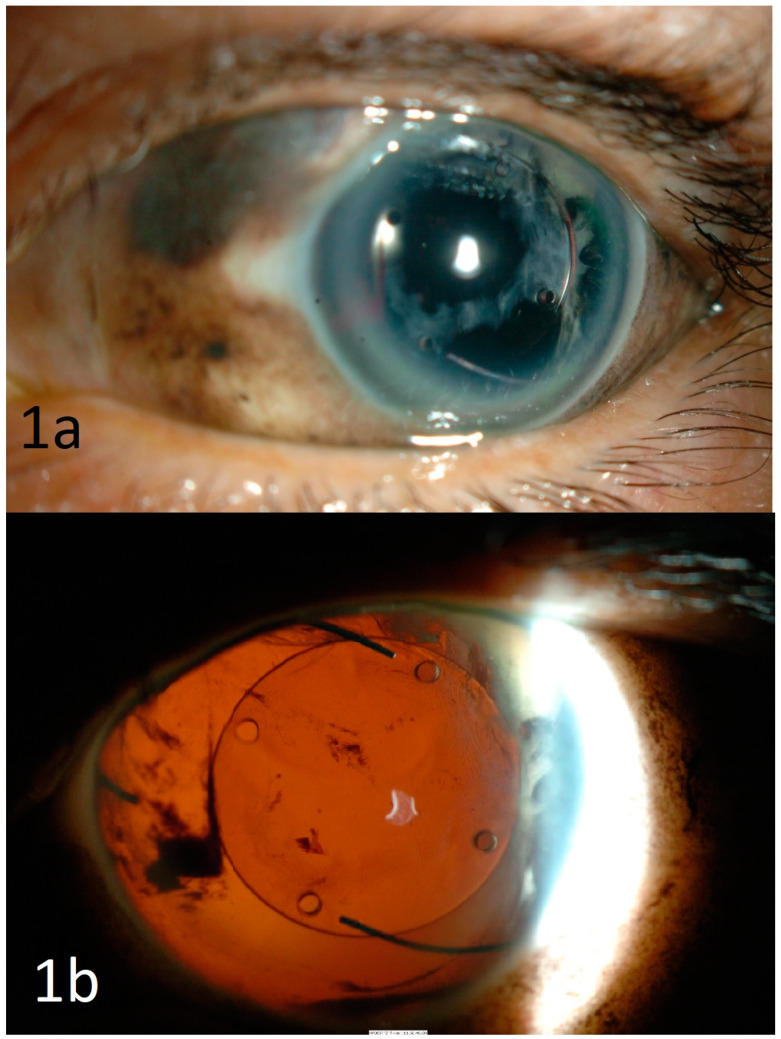
Slit-lamp examination photographs. (**a**) Slit-lamp examination photograph of the left (injured) eye showing loss of iris and iris pigments found under the subconjunctival space superonasally, with a well-centred intraocular lens in the posterior capsular bag with no subluxation. (**b**) Slit-lamp examination photograph of the left (injured) eye a month after the injury showing complete loss of iris.

A 71-year-old lady was admitted to the hospital following a mechanical fall down one flight of stairs. She was known to be pseudophakic in both eyes (BE) from routine uncomplicated cataract surgeries in the 1980s. On examination, there was periocular haematoma, subconjunctival haemorrhage, hyphaema, and vitreous haemorrhage in the left eye. Her visual acuity (VA) at presentation was 6/6 in the right eye (RE) and 6/18 in the left eye (LE). The cornea was intact and the intraocular pressures were normal in BE. As there was no fundal view, B-scan ultrasonography of the injured eye was performed, which showed flat retina with no evidence of globe rupture. Five days later, with reducing hyphaema and vitreous haemorrhage, it was noted that there was complete loss of iris in the anterior segment, with possible iris pigments found in the subconjunctival space superonasally (Figure 1a). The intraocular lens was well-centred in the posterior capsular bag with no subluxation. Fundus examination were unremarkable. Patient was treated with tapering regime of topical steroid eye drops, mydriatics, and oral ciprofloxacin. 

**Figure 2:**
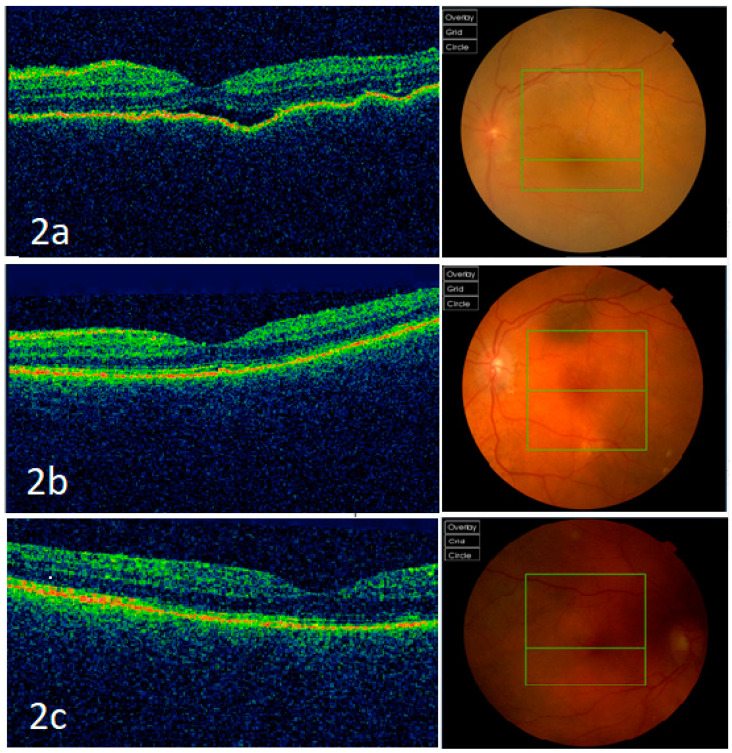
Optical coherence tomography (OCT) macula and fundus photographs. (**a**) Optical coherence tomography (OCT) macula of the left eye showing retinal pigment epithelium irregularities and some subretinal fluid at presentation of sympathetic ophthalmia, and its corresponding fundus photograph. (**b**) OCT macula and the corresponding fundus photograph of the left eye three months after commencing treatment, showing normal appearances. (**c**) OCT macula and the corresponding fundus photograph of the right eye three months after commencing treatment, showing normal appearances.

A month later, the patient was re-admitted to the hospital for acute confusion secondary to a urinary tract infection and reduced vision in BE. There was no history of further trauma to the eyes. On examination, the VA was 6/12 in the RE and counting finger in the LE. There was mild conjunctival injection, keratic precipitates, anterior chamber flare, 180-degree posterior synechiae, and vitritis with no fundal view in the RE. There were some anterior chamber cells with loss of iris in the LE (Figure 1b). Optical coherence tomography (OCT) macula of the LE showed retinal pigment epithelium irregularities and some subretinal fluid (Figure 2a). The macula OCT could not be obtained for the right eye due to poor fundal view secondary to vitritis. Although there was no further history of trauma and the globe was intact, she was diagnosed with sympathetic ophthalmia given the uveal tissue disruption in the form of complete iris disinsertion. Further blood tests such as serum angiotensin converting enzyme, Treponemal serology, and QuantiFERON-TB Gold were negative. She was commenced on intravenous methylprednisolone, and then reducing doses of oral steroid. Vitritis of the right eye settled and there were no signs of vasculitis on fundal examination. The OCT macula of the LE and RE three months after commencing treatment were normal, as shown in Figure 2b,c, respectively. The VA improved to 6/6 in the RE and 6/9 in the LE. She remained stable on oral cyclosporine with no recurrence. 

## 3. Discussion

Diagnosis of sympathetic ophthalmia is often challenging, and is usually based on history and clinical examination findings [11]. It classically presents as bilateral granulomatous panuveitis with a definitive history of iatrogenic or non-iatrogenic penetrating injury to the eye [12]. It is usually accompanied with moderate to dense vitritis, choroiditis, and papillitis with multiple exudative retinal detachments [13]. The aetiology and pathogenesis of sympathetic ophthalmia remains unclear [14]. It has been suggested that the bilateral granulomatous hypersensitivity reaction is T-cell mediated and is triggered by inciting antigens, such as S antigen or Mart-1 melanoma antigen directed against exposed uveal tissue in the injured (exciting) eye, typically following penetrating eye trauma or intraocular surgery that involves manipulation of uveal tissue [2,13].

Our case emphasises the importance of considering sympathetic ophthalmia as a differential diagnosis in cases of unexplained panuveitis even after blunt trauma. As in our case, the only striking feature is the loss of iris of the injured eye. The initial diagnosis of sympathetic ophthalmia was challenging due to the lack of history of penetrating eye injury and the fact that the loss of iris was only found when hyphaema was settling. Several considerations were given before the diagnosis of sympathetic ophthalmia was made in our patient. Firstly, we noted that she was pseudophakic in BE. This immediately excluded the diagnosis of Vogt–Koyanagi–Harada disease even though it is an autoimmune condition that shares similar immunologic mechanisms and clinical features with sympathetic ophthalmia. Sympathetic ophthalmia can develop in variable time intervals after the inciting event, with its occurrence after surgically-induced cases (median 14.3 months) being later than that of ocular trauma (median 6.5 months) [15]. In our case, sympathetic ophthalmia occurred approximately 6.5 weeks after the blunt trauma; therefore, given the recent event, we believe that the previous uncomplicated cataract surgery was unlikely to be the cause. Other differential diagnoses such as sarcoidosis and infective aetiologies of syphilis and tuberculosis were also excluded.

Development of sympathetic ophthalmia in non-penetrating trauma is very uncommon. We report an interesting presentation of clinically diagnosed sympathetic ophthalmia after blunt trauma with no evidence of penetrating eye injury. To our knowledge, there has been only one case report published in 2009 describing sympathetic ophthalmia following a blunt trauma caused by a bungee cord, which has led to hyphaema but not an open globe injury [16]. This case certainly concurs with our case in that non-penetrating injury can also result in sympathetic ophthalmia. However, there has been no report of complete disinsertion of iris in an intact globe as its cause in the current literature. Another relevant case report by Khatri et al. described a case of sympathetic ophthalmia caused by a blunt trauma to the phthisical eye, which suffered a penetrating eye injury 17 years back [17]. The authors suggested that damage to certain uveal tissues may itself be sufficient to trigger an immune response and a penetrating trauma may not be a prerequisite for the development of sympathetic ophthalmia [17,18]. Our case report supports this hypothesis and indeed, sympathetic ophthalmia has also been reported in association with keratitis [4,5], chemical burns [2], and insect sting [19], in the absence of penetrating eye injury or any intraocular procedures.

The patient in our case was treated with intensive systemic and topical corticosteroid therapy, which by and large gives good prognosis with regular follow-up and timely treatment. Immunomodulating agents were initiated for steroid sparing effects [20]. It is conventionally accepted that enucleation of the injured eye should be performed to prevent sympathetic ophthalmia [21]. However, it is important to note that enucleation cannot prevent further recurrences in the sympathising eye [22]. We also found it hard to justify enucleation in eyes with non-penetrating trauma with remaining useful vision as in this case, in order to prevent sympathetic ophthalmia. Furthermore, corticosteroids were introduced early in our case, and there was significant clinical improvement with treatment.

In conclusion, we have described an atypical presentation of sympathetic ophthalmia following a non-penetrating ocular trauma. Loss of iris in this case is an interesting ocular finding that has not been previously reported. Ophthalmologists should be mindful that non-penetrating ocular trauma can still cause sympathetic ophthalmia, and a thorough history of the mechanism of injury and comprehensive ophthalmic examination are essential so that prompt treatment can be provided to improve the visual prognosis of affected patients.

## Data Availability

No new data were created or analyzed in this study. Data sharing is not applicable to this article.
